# Construction of a predictive model for blood transfusion in patients undergoing total hip arthroplasty and identification of clinical heterogeneity

**DOI:** 10.1038/s41598-024-51240-2

**Published:** 2024-01-06

**Authors:** Jicai Deng, Chenxing Zhou, Fei Xiao, Jing Chen, Chunlai Li, Yubo Xie

**Affiliations:** 1 Department of Anesthesiology, The First Affiliated Hospital of Guangxi Medical University, Nanning, 530021 Guangxi People’s Republic of China; 2 Department of Anesthesiology, The Fifth Affiliated Hospital of Guangxi Medical University, Nanning, Guangxi People’s Republic of China; 3 Department of Spine and Osteopathy Ward, The First Affiliated Hospital of Guangxi Medical University, Nanning, Guangxi People’s Republic of China; 4 Guangxi Key Laboratory of Enhanced Recovery After Surgery for Gastrointestinal Cancer, The First Affiliated Hospital of Guangxi Medical University, Nanning, Guangxi People’s Republic of China

**Keywords:** Computational biology and bioinformatics, Machine learning, Predictive markers

## Abstract

A precise forecast of the need for blood transfusions (BT) in patients undergoing total hip arthroplasty (THA) is a crucial step toward the implementation of precision medicine. To achieve this goal, we utilized supervised machine learning (SML) techniques to establish a predictive model for BT requirements in THA patients. Additionally, we employed unsupervised machine learning (UML) approaches to identify clinical heterogeneity among these patients. In this study, we recruited 224 patients undergoing THA. To identify factors predictive of BT during the perioperative period of THA, we employed LASSO regression and the random forest (RF) algorithm as part of supervised machine learning (SML). Using logistic regression, we developed a predictive model for BT in THA patients. Furthermore, we utilized unsupervised machine learning (UML) techniques to cluster THA patients who required BT based on similar clinical features. The resulting clusters were subsequently visualized and validated. We constructed a predictive model for THA patients who required BT based on six predictive factors: Age, Body Mass Index (BMI), Hemoglobin (HGB), Platelet (PLT), Bleeding Volume, and Urine Volume. Before surgery, 1 h after surgery, 1 day after surgery, and 1 week after surgery, significant differences were observed in HGB and PLT levels between patients who received BT and those who did not. The predictive model achieved an AUC of 0.899. Employing UML, we identified two distinct clusters with significantly heterogeneous clinical characteristics. Age, BMI, PLT, HGB, bleeding volume, and urine volume were found to be independent predictors of BT requirement in THA patients. The predictive model incorporating these six predictors demonstrated excellent predictive performance. Furthermore, employing UML enabled us to classify a heterogeneous cohort of THA patients who received BT in a meaningful and interpretable manner.

## Introduction

Total hip arthroplasty (THA) is a common surgical intervention for treating various hip joint diseases, including osteonecrosis of the femoral head, hip ankylosis caused by ankylosing spondylitis, and hip osteoarthritis^1,2^. Effective blood management is a critical component of the perioperative care of THA patients^3^. Previous studies have indicated that blood transfusion is independently linked to higher morbidity and mortality in THA patients^4,5^. Strategies aimed at reducing perioperative blood loss and minimizing the need for allogeneic red blood cell transfusions encompass various measures, including addressing preoperative anemia^6^, administering anti-fibrinolytic therapy^7^, and utilizing intraoperative cell salvage techniques^8,9^. Effective implementation of the aforementioned measures relies on our ability to accurately predict the need for perioperative blood transfusion (BT) in THA patients. It is crucial to develop a precise predictive model to forecast the need for perioperative BT in THA, which holds significant clinical value. Furthermore, if BT events occur during the perioperative period of THA patients, clinicians need to pay closer attention to the perioperative blood management of such patients, preventing adverse events arising from hemodynamic abnormalities. Therefore, it is crucial to identify clinical heterogeneity among THA patients requiring transfusion and ascertain the presence of clusters with significantly characteristic risks among this patient population. This is essential for comprehensive perioperative blood management and the prevention of adverse events in patients undergoing THA.

In recent times, the rapid advancement of artificial intelligence has led to the increased application of machine learning (ML), a subfield of AI, in disease classification, diagnosis, and treatment. Notably, ML has been employed in addressing conditions like heart failure and pediatric dermatitis^10^. Precision medicine has emerged as a leading approach in modern medical practice, offering improved medical efficiency and reduced incidence of complications during medical procedures^11^. Precision medicine necessitates the precise identification and classification of patients, followed by the implementation of distinct medical interventions tailored to each patient's specific needs^10^. The incorporation of artificial intelligence and ML algorithms has ushered in a new era for precision medicine. ML comprises two principal categories, namely supervised machine learning (SML) and unsupervised machine learning (UML). SML employs large accurately labeled training datasets and iterative algorithms^12^; UML aims to cluster patients based on their clinical features. The integration of SML and UML techniques enables the identification of specific categories and the grouping of patients, facilitating the analysis of characteristics among similarly clustered individuals. This approach may aid in the identification of novel disease subtypes and accelerate the adoption of precision medicine.

In this study, we gathered clinical data from THA patients and utilized SML to establish a predictive model for perioperative blood transfusion requirements in these patients. Independent predictors linked to transfusion requirements were identified. We subsequently employed UML to classify THA patients who received blood transfusions during the perioperative period, ultimately identifying two distinct clusters. Finally, we conducted a differential analysis to explore the heterogeneity of these clusters. The study aims to integrate both supervised and unsupervised machine learning techniques to enhance perioperative blood management in patients undergoing THA. This integration aims to enable timely medical interventions by clinicians, mitigating the occurrence of adverse events during the perioperative period.

## Materials and methods

### Patients and data collection

**Figure 1:**
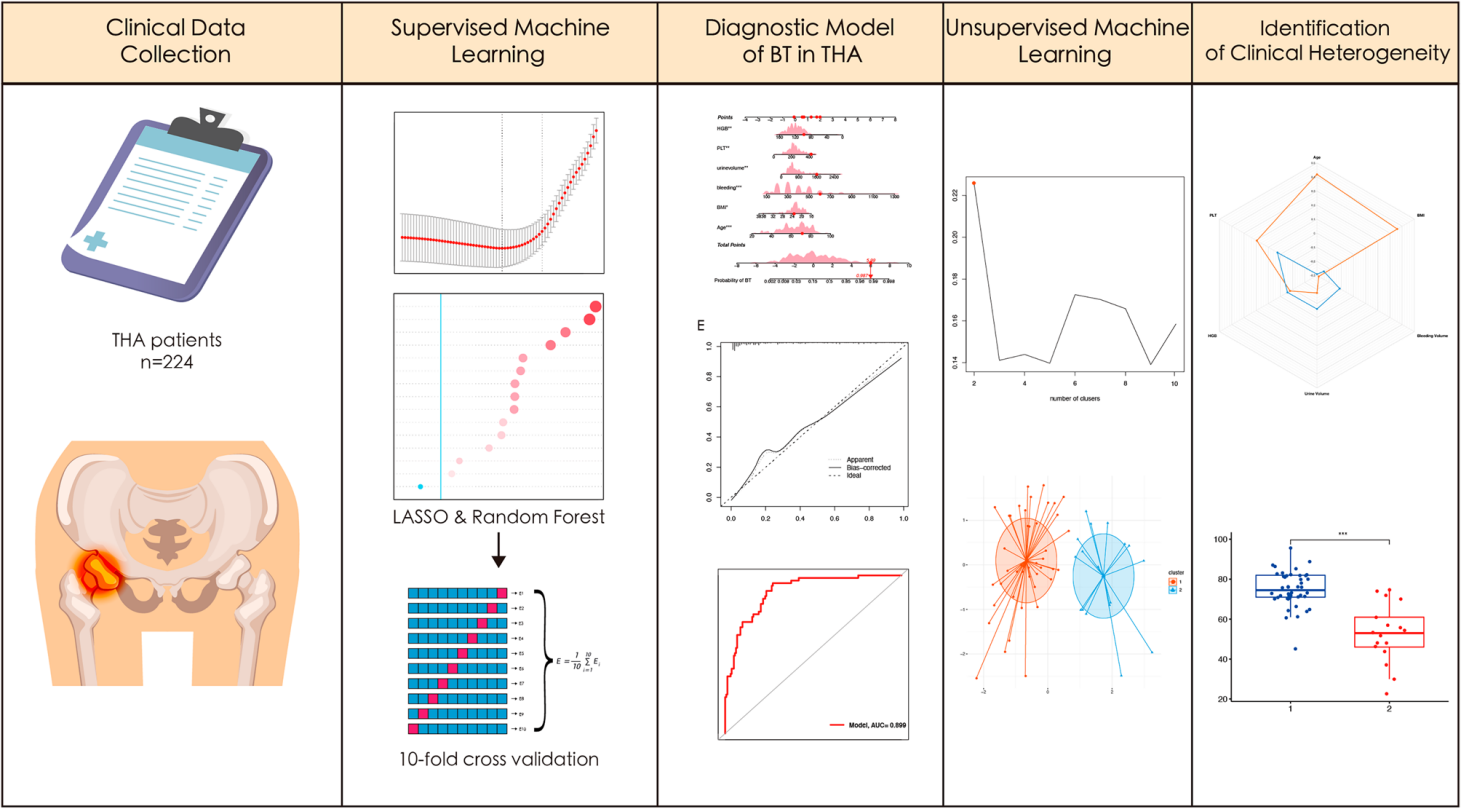
The graphical abstract of this study.

We conducted a retrospective analysis of clinical data (Table 1) collected from 224 patients who underwent total hip arthroplasty at the First People's Hospital of Nanning between 2015 and 2022. Figure 1 shows the graphical abstract of this study. Inclusion criteria were (1) age ≥ 18 years old; (2) ASA Grade II-IV^13^; (3) Unilateral, total hip arthroplasty had been performed. Exclusion criteria were (1) patients undergoing revision hip surgery, and (2) patients undergoing, bilateral hip replacement at the same time. We collected 44 perioperative variables in the clinical data, which were age, gender, body mass index (BMI), American Society of Anesthesiologists (ASA) grade, hypertension, pulmonary infection, diabetes, cerebral infarction, cardiovascular disease (CVD), chronic obstructive pulmonary disease (COPD), renal failure (RF), pulmonary arterial hypertension (PAH), hip fracture, operation time, bleeding volume, autotransfusion, tranexamic acid (TXA), anesthesia method, systolic pressure (SP), diastolic pressure (DP), heart rate (HR), SpO_2_, colloid, crystalloid, urine volume, drainage volume, red blood cell (RBC), Hemoglobin (HGB), hematocrit (HCT), platelet (PLT), total bilirubin (TBIL), total protein (TP), albumin (ALB), alanine aminotransferase (ALT), aspartate aminotransferase (AST), creatinine (CREA), blood urea nitrogen (BUN), cystatin C (Cys-C), creatinine clearance rate (Ccr), hypersensitive C-reactive protein (hs-CRP), prothrombin time (PT), activated partial thromboplastin time (APTT), fibrinogen (FIB), and D-dimer (DD). Additionally, postoperative observations and data collection were undertaken to gather clinical information from patients at 1 h, 1 day, and 1 week following total hip arthroplasty. The clinical data encompassed measurements of RBC, HGB, HCT, PLT, TBIL, TP, ALB, ALT, AST, CREA, BUN, Cys-C, Ccr, Hs-CRP, PT, APTT, FIB, and DD.

**Table 1:** Baseline characteristics between BT patients and non-BT patients.

Clinical Characteristics	Overall	BT	Non-BT	*P*-Value
	(n = 224)	(n = 61)	(n = 163)	
Age				< 0.001
Mean ± SD	64.16 ± 13.837	68.84 ± 14.66	62.41 ± 13.14	
Median [P25, P75]	66 [55, 74]	72 [61, 80]	63 [54, 72]	
Gender				0.900
Male	127 (56.6%)	35 (57.3%)	92 (56.4%)	
Female	97 (43.4%)	26 (42.7%)	71 (43.5%)	
BMI				0.005
Mean ± SD	22.73 ± 2.94	21.82 ± 2.42	23.07 ± 3.04	
Median [P25, P75]	22.58 [20.81, 24.03]	22.19 [19.78, 23.07]	22.81 [21.26, 24.24]	
ASA				0.383
II	97 (43.30%)	24 (39.34%)	73 (44.79%)	
III	121 (54.01%)	34 (55.74%)	87 (53.37%)	
IV	6 (2.68%)	3 (4.92%)	3 (1.84%)	
Hypertension				0.530
	70 (31.25%)	21 (34.43%)	49 (30.06%)	
Pulmonary Infection				0.841
	10 (4.46%)	3 (5.17%)	7 (4.29%)	
Diabetes				0.711
	21 (9.38%)	5 (8.93%)	16 (9.82%)	
Cerebral Infarction				0.248
	12 (5.36%)	5 (8.93%)	7 (4.29%)	
CVD				0.022
	8 (3.57%)	5 (8.93%)	3 (1.84%)	
COPD				0.936
	7 (3.12%)	2 (3.28%)	5 (3.07%)	
RF				0.101
	1 (0.45%)	1 (1.63%)	0 (0.00%)	
PAH				0.467
	2 (0.89)	1 (1.63%)	1 (0.61%)	
Fracture				0.437
	108 (48.21%)	32 (52.46%)	76 (46.63%)	
Operation Time				0.092
Mean ± SD	125.27 ± 28.24	128.18 ± 25.2	124.18 ± 29.29	
Median [P25, P75]	120 [105, 140]	129 [113.5, 143.5]	120 [104, 140]	
Bleeding Volume				< 0.001
Mean ± SD	369.42 ± 184.40	509.84 ± 202.04	316.87 ± 146.59	
Median [P25, P75]	300 [200, 500]	500 [400, 600]	300 [200, 400]	
Autotransfusion				< 0.001
Mean ± SD	91.96 ± 153.88	159.02 ± 175.72	66.87 ± 137.24	
Median [P25, P75]	100 [0, 250]	200 [0, 250]	200 [0, 200]	
TXA				< 0.001
0 g	58 (25.89%)	27 (44.26%)	31 (19.02%)	
1 g	117 (52.23%)	27 (44.26%)	90 (55.21%)	
2 g	49 (21.88%)	7 (11.48%)	42 (25.77%)	
Anesthesia Method				0.081
General Anesthesia	14 (6.25%)	1 (1.63%)	13 (7.98%)	
SP				0.733
Mean ± SD	146.32 ± 18.617	147.02 ± 20.65	146.06 ± 17.86	
Median [P25, P75]	146 [133.25, 158.75]	150 [134, 164]	146 [132, 158]	
DP				0.551
Mean ± SD	85.74 ± 26.97	83.49 ± 11.71	86.58 ± 30.79	
Median [P25, P75]	84.5 [77, 91]	82 [77.5, 92]	85 [77, 91]	
HR				0.002
Mean ± SD	80.37 ± 12.10	83.39 ± 12.09	79.24 ± 11.94	
Median [P25, P75]	79 [72, 88]	85 [76, 90]	78 [70, 86]	
SpO_2_				0.170
Mean ± SD	97.11 ± 2.16	97.28 ± 2.45	97.05 ± 2.04	
Median [P25, P75]	97.5 [96, 99]	98 [96, 99]	97 [96, 99]	
Colloid				0.002
Mean ± SD	681.47 ± 340.84	804.92 ± 404.63	635.28 ± 302.30	
Median [P25, P75]	500 [500, 1000]	750 [500, 1000]	500 [500, 950]	
Crystalloid				0.463
Mean ± SD	1156.25 ± 407.20	1120.16 ± 470.48	1169.75 ± 381.56	
Median [P25, P75]	1150 [850, 1400]	1100 [850, 1400]	1200 [900, 1400]	
Urine Volume				0.013
Mean ± SD	636.96 ± 360.18	764.43 ± 465.74	589.26 ± 299.73	
Median [P25, P75]	500 [400, 800]	600 [425, 875]	500 [400, 700]	
Drainage Volume				0.414
Mean ± SD	304.57 ± 180.06	326.43 ± 198.99	296.38 ± 172.39	
Median [P25, P75]	280 [170, 400]	300 [170, 425]	275 [170, 400]	
RBC				0.008
Mean ± SD	4.36 ± 2.60	4.02 ± 0.63	4.48 ± 3.02	
Median [P25, P75]	4.11 [3.71, 4.56]	4.01 [3.68, 4.29]	4.24 [3.84, 4.63]	
HGB				< 0.001
Mean ± SD	120.13 ± 20.47	111.65 ± 17.40	123.30 ± 20.67	
Median [P25, P75]	120 [107, 133]	111[98.5,124]	125 [111, 137]	
HCT				< 0.001
Mean ± SD	36.68 ± 21.61	38.21 ± 38.02	36.11 ± 10.30	
Median [P25, P75]	37.45 [32.83, 40.5]	34.6 [30.15, 38.6]	38.07 [34.6, 41.2]	
PLT				0.017
Mean ± SD	242.28 ± 77.45	263.23 ± 82.72	234.44 ± 74.13	
Median [P25, P75]	229 [195.5, 284.75]	250 [211, 318]	225 [190, 268]	
TBIL				0.398
Mean ± SD	12.45 ± 14.18	10.91 ± 6.55	13.03 ± 16.11	
Median [P25, P75]	10.1 [7.43, 14.23]	10 [6.95, 13.05]	10.1 [7.4, 14.7]	
TP				0.184
Mean ± SD	63.75 ± 7.06	64.78 ± 7.88	63.36 ± 6.74	
Median [P25, P75]	63.67 [58.74, 68.58]	64.7 [59.1, 69.1]	63.3 [58.2, 67.8]	
ALB				0.456
Mean ± SD	36.66 ± 5.03	36.25 ± 5.40	36.81 ± 4.90	
Median [P25, P75]	36.83 [32.83, 39.8]	37.1 [33.2, 40.1]	36 [21.15, 38.55]	
ALT				0.207
Mean ± SD	20.93 ± 18.38	24.07 ± 23.73	19.52 ± 15.79	
Median [P25, P75]	16^11,23^	17^11,29^	15^11,21^	
AST				0.607
Mean ± SD	24.17 ± 13.31	25.72 ± 15.35	23.59 ± 12.64	
Median [P25, P75]	20^16,27^	20 [16, 29.25]	19^16,27^	
CREA				0.404
Mean ± SD	79.02 ± 23.74	84.09 ± 31.08	77.12 ± 20.11	
Median [P25, P75]	74.5 [63, 92]	76 [63, 101]	74 [63, 91]	
BUN				0.237
Mean ± SD	5.51 ± 2.55	5.82 ± 2.69	5.39 ± 2.50	
Median [P25, P75]	5 [3.9, 6.40]	5.2 [4.08, 7.1]	4.93 [3.9, 6.24]	
Cys-C				0.021
Mean ± SD	1.22 ± 0.71	1.35 ± 0.83	1.17 ± 0.66	
Median [P25, P75]	1.07 [0.95, 1.27]	1.06 [0.94, 1.23]	1.18 [0.96, 1.58]	
Ccr				0.029
Mean ± SD	71.03 ± 20.47	65.99 ± 23.21	72.92 ± 19.09	
Median [P25, P75]	71.48 [59.04, 82.08]	72.2 [61.54, 82.08]	62.97 [46.3, 84.18]	
hs-CRP				0.135
Mean ± SD	31.38 ± 38.66	35.21 ± 37.59	29.95 ± 39.07	
Median [P25, P75]	16.19 [4.30, 44.85]	22.92 [6.75, 56.11]	15.82 [3.9, 42.6]	
PT				0.264
Mean ± SD	13.14 ± 1.05	13.01 ± 1.15	13.18 ± 1.01	
Median [P25, P75]	13.2 [12.43, 13.8]	13.1 [12.3, 13.65]	13.2 [12.5, 13.8]	
APTT				0.687
Mean ± SD	35.87 ± 5.28	36.16 ± 5.77	35.76 ± 5.10	
Median [P25, P75]	35.85 [32.15, 39.4]	36.3 [31.35, 40.1]	35.8 [32.6, 39.1]	
FIB				0.285
Mean ± SD	4.17 ± 1.27	4.37 ± 1.41	4.10 ± 1.21	
Median [P25, P75]	3.98 [3.17, 4.96]	4.22 [3.13, 5.34]	3.92 [3.17, 4.82]	
DD				0.426
Mean ± SD	4.49 ± 6.41	5.40 ± 7.93	4.15 ± 5.73	
Median [P25, P75]	2.35 [0.97, 5.03]	2.54 [1, 6.5]	2.18 [0.96, 4.86]

The Ethics Committee of The Fifth Affiliated Hospital of Guangxi Medical University reviewed and approved the study. All patients provided informed consent and willingly participated in the study. The clinical data involved in this study has obtained explicit authorization from the patients. The study complies with the Declaration of Helsinki.

### Statistical analysis of clinical data

Concerning clinical data with missing values, we employed the expectation maximization method in SPSS Version 22.0 for imputation. The original dataset and the data with imputed missing values are available in the supplementary materials. Clinical data were presented as Mean ± SD and Median [P25, P75]. We performed statistical analyses using SPSS version 22.0, employing the Mann–Whitney U test, Student's t-test, or chi-square test as appropriate. These tests were employed to compare disparities between patients with and without blood transfusions (BT and Non-BT), depending on the data type. The significance level was set at α = 0.05. To establish a predictive model for blood transfusion in THA patients, we employed the logistic regression algorithm and created a nomogram to visualize the prediction model. The 'corrplot' package in R software was used to generate correlation heat maps illustrating the correlation between clinical data in the prediction model. The accuracy of the prediction model was determined by the ROC curve and calibration curve analyses (The 'pROC' package in R software).

### LASSO-regularized linear regression

The Least Absolute Shrinkage and Selection Operator (LASSO) regression is a contraction algorithm developed to manage variables with multicollinearity. It streamlines the process of parameter estimation and generates sparse solutions, enabling efficient variable selection. Consequently, it is an appropriate method for addressing multicollinearity problems and improving test efficiency^14–16^. In this study, clinical data showing significant differences (*p* < 0.05) were entered into the R software to perform LASSO regression using the 'glmnet' package, which aimed to identify factors that could predict the necessity for blood transfusion in THA patients. To avoid overfitting, we employed ten rounds of tenfold cross-validation^15^.

### Random forest

We used the "randomforest" package in R software to screen clinical data by employing the Random Forest algorithm^17^. The Random Forest algorithm works by assigning random values to each clinical characteristic. If a characteristic is considered more important, randomly changing its value will lead to a higher prediction error for the model^18^. The clinical characteristics become more significant as their value increases.

### Development of a logistic regression-based predictive model

Following the initial clinical characteristics screening via supervised machine learning (SML), we proceeded to utilize univariate logistic regression analysis for the evaluation of the association between blood transfusion (BT) necessity in total hip arthroplasty (THA) patients and their clinical characteristics. Variables with p-values below 0.05 were included in the subsequent multivariate logistic regression analysis. Based on the outcomes of the multivariate logistic regression analysis, which incorporated variables with *p*-values less than 0.05, we constructed a predictive model for assessing the need for blood transfusion in THA patients, represented using a nomogram. Model performance was evaluated in terms of discrimination and calibration^19^. Calibration of the prediction model involved the creation of a visual calibration plot, which compared predicted and actual probabilities of blood transfusion (BT) requirement. The model's discriminative capability was assessed through the Area under the Curve (AUC) of the Receiver Operating Characteristic (ROC) curve, which varies from 0.5 (indicating no discrimination) to 1 (indicating perfect discrimination)^20^.

### Unsupervised machine learning for clinical heterogeneity identification

To ascertain whether there are distinct risk-characterized clusters among patients requiring BT in THA, we employed UML to further identify clinical heterogeneity. To conduct UML, we used R software version 4.1.3. We normalized the clinical data of THA patients who received BT during the perioperative period (n = 61) by utilizing the Scale Function in the "factoextra" package^21^. To determine the optimal clustering number (K value), we used the "Fpc" package, which utilizes the Silhouette Coefficient (SC)^11,22,23^. The K-means clustering algorithm is a well-known unsupervised learning technique in machine learning. In this study, we utilized the K-means algorithm to group patients into clusters^23–25^. The clusters derived from the K-means algorithm were visually presented using clustergram and radargram.

The K-means algorithm is an Unsupervised Machine Learning algorithm that can categorize and identify data^11^. The K-means clustering algorithm can effectively group clinical data based on their characteristics, even if their labels are unknown. These groups, or "clusters," are represented by a central point called a "centroid." To initiate the clustering process, the clinical data is normalized using the Scale Function, and the K-means algorithm is applied through the following steps: (I) K initial centroids are randomly chosen, and each sample point is assigned to the nearest centroid to form K clusters. (II) A new centroid is calculated for each cluster by computing the average coordinate value of all points within the cluster. (III) This process is repeated until the position of the centroids remains stable. The optimal value of K is determined in this study using the Silhouette Coefficient (SC).

$$SC(i)=\frac{{\text{b}}\left({\text{i}}\right)-{\text{a}}({\text{i}})}{{\text{max}}[{\text{a}}\left({\text{i}}\right),{\text{b}}\left({\text{i}}\right)]}$$

The formula employed in this study uses a(i) to represent the average distance between a sample point and all other points within the same cluster. b(i), on the other hand, refers to the average distance between the sample point and all points within the second closest cluster^26^. The main objective of the K-means clustering algorithm is to decrease the within-cluster variation and increase the between-cluster variation. The Silhouette Coefficient is used to assess the quality of clustering, with values ranging from –1 to 1. A higher value closer to 1 suggests better clustering performance, while a value closer to –1 indicates poor results.

We employed the K-means algorithm to cluster patients undergoing THA who received BT based on six independent predictive factors from the predictive model, aiming to identify clinical heterogeneity. We employed radargram for visualizing the heterogeneity between the two patient clusters and compared differences in Age, BMI, HGB, PLT, Bleeding Volume, and Urine Volume. Additionally, box plots were used to visually represent the data variances, offering an intuitive presentation of the clinical heterogeneity among patients.

### Ethical approval

The study got approval by Ethics Department of the Fifth Affiliated Hospital of Guangxi Medical University (No. 2021-064-01). All subjects of this study are volunteered and signed informed consent forms. The clinical data involved in this study has obtained explicit authorization from the patients. The study complies with the Declaration of Helsinki.

## Result

### Results of SML: tenfold cross-validation LASSO regression and random forest

Table 1 presents the clinical data of 224 patients who underwent total hip arthroplasty. Among them, 61 patients received blood transfusions (BT) during the perioperative period, while 163 did not. Statistical significance (*P* < 0.05) was observed in 15 clinical characteristics between the BT and non-BT patients, including Age, BMI, CVD, Bleeding volume, autotransfusion, TXA, HR, colloid, urine volume, RBC, HGB, HCT, PLT, Cys-C, and Ccr.

**Figure 2:**
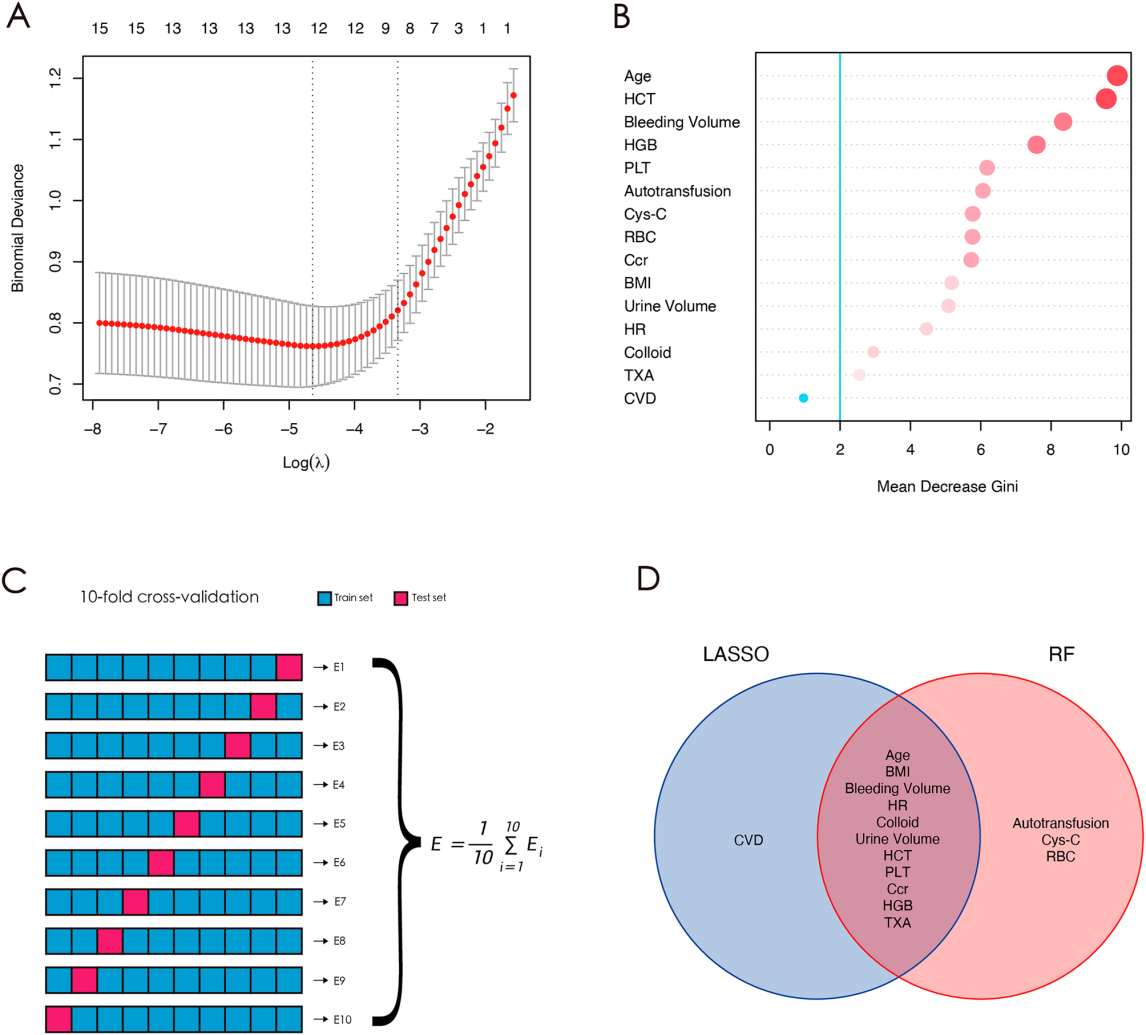
The results of LASSO regression (**A**). 14 predictive factors were screened by Random Forest algorithms (**B**). The pattern diagram for five iterations of tenfold cross validation (**C**). Intersection of predictive factors screened using LASSO regression and Random Forest algorithm (**D**).

To identify predictive factors of perioperative blood transfusion, LASSO regression analysis was conducted on the clinical data with significant differences. The results are shown in Supplementary Fig. 1A and Fig. 2A, which displays the 12 predictive factors of perioperative blood transfusion: Age, BMI, Bleeding volume, HR, colloid, urine volume, HCT, PLT, Ccr, HGB, CVD, and TXA.

In a similar vein, we utilized the Random Forest algorithm to identify predictive factors for patients with BT during the perioperative period by analyzing the clinical data with significant differences in Table 1. We set the number of decision trees to 20,000 and observed that the error rate of the model became stable (Supplementary Fig. 1B). From the eight clinical characteristics, we selected the top 14 factors with the highest importance (Fig. 2B). These factors were Age, HCT, Bleeding Volume, HGB, PLT, Autotransfusion, Cys-C, RBC, Ccr, BMI, Urine Volume, HR, Colloid, and TXA.

To avoid overfitting, we conducted ten rounds of tenfold cross-validation for the outcomes of LASSO regression and Random Forest algorithm. Figure 2C demonstrates the graphical representation of this approach. The intersection of the 11 predictors identified through LASSO regression and Random Forest algorithm are Age, BMI, Bleeding Volume, HR, Colloid, Urine Volume, HCT, PLT, Ccr, HGB, and TXA, as shown in Fig. 2D.

### Construction of a prediction model for blood transfusion in patients undergoing THA

**Figure 3:**
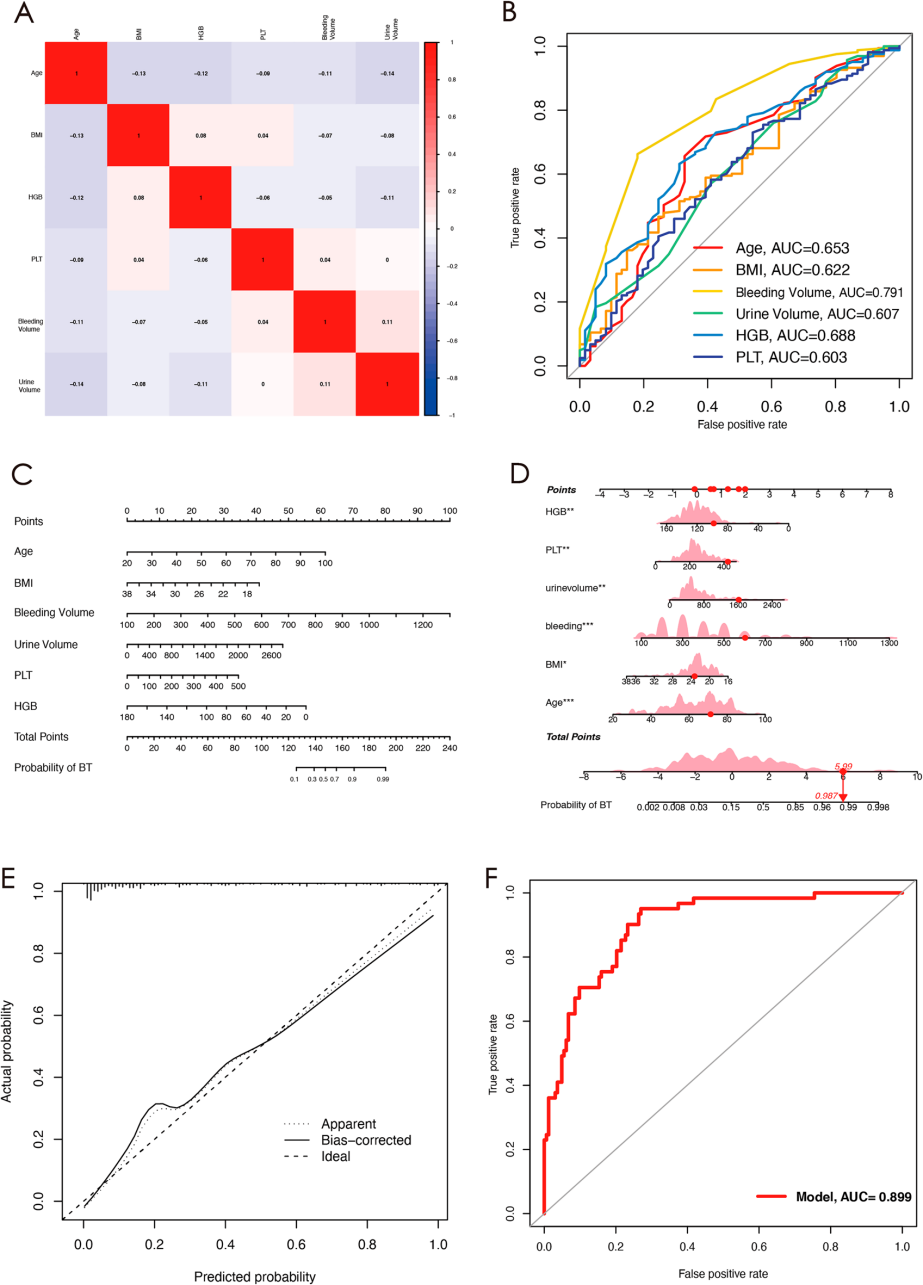
Six independent predictors were evaluated using univariate and multivariate logistic regression. Red color piece indicates a positive correlation, while blue color piece indicates a negative correlation. The strength of the correlation increases with the increase in color intensity (**A**). The diagnostic ability of six clinical characteristics was assessed using ROC curves in the clinical data (**B**). Nomogram for predicting of patient who received BT (**C**). A nomogram represents a BT patient in THA. Red dots indicate the patient's score and total score for each clinical characteristic, and arrows indicate the probability BT (**D**). Calibration curves for predicting a BT patient in THA (**E**). AUC of the nomogram (**F**).

In this research study, we conducted univariate and multivariate logistic regression analyses on 11 factors to develop a clinical prediction model for patients who underwent total hip arthroplasty with perioperative blood transfusion. Table 2 presents the outcomes of the univariate and multivariate logistic regression, which showed that six independent variables were selected as predictors, namely Age, Body Mass Index (BMI), Hemoglobin (HGB), Platelet count (PLT), Bleeding Volume, and Urine Volume. A heatmap in Fig. 3A illustrates the correlation between these six independent variables. The area under the curve (AUC) values for each of these six independent variables in predicting the need for blood transfusion were 0.653 for age, 0.622 for BMI, 0.688 for HGB, 0.603 for PLT, 0.791 for Bleeding Volume, and 0.607 for Urine Volume (Fig. 3B). A nomogram was utilized to visualize the prediction model based on these six independent variables (Fig. 3C), which displays a sample of a patient who underwent total hip arthroplasty and required perioperative blood transfusion (Fig. 3D). Calibration curves were generated to validate the accuracy of the nomogram's predicted probabilities, and these curves revealed a satisfactory level of agreement between the predicted and actual probabilities (Fig. 3E). The ROC curve for the nomogram is shown in Fig. 3F, with an AUC value of 0.899.

**Table 2:** Univariate and multivariate logistic regression used for identifying independent diagnostic factors to distinguish BT patients from Non-BT patients.

Clinical characteristics	Univariate logistic regression		Multivariate logistic regression	
	OR [95%CI]	P-value	OR [95%CI]	P-value
Age	1.082 [1.039, 1.128]	< 0.001	0.924 [0.892, 0.958]	< 0.001
BMI	0.790 [0.655, 0.952]	0.013	1.210 [1.020, 1.435]	0.029
Bleeding Volume	1.008 [1.005, 1.011]	< 0.001	0.992 [0.989, 0.994]	< 0.001
HR	1.022 [0.988, 1.057]	0.213		
Colloid	1.001 [1.000, 1.003]	0.165		
Urine Volume	1.002 [1.000, 1.003]	0.007	0.998 [0.997, 0.999]	0.004
HCT	1.013 [0.992, 1.034]	0.238		
PLT	1.007 [1.001, 1.013]	0.018	0.993 [0.988, 0.998]	0.010
CCR	0.988 [0.967, 1.010]	0.294		
HGB	0.961 [0.939, 0.983]	0.001	1.032 [1.011, 1.053]	0.003
TXA	1.038 [0.240, 4.497]	0.988

### Postoperative observation of independent predictors of blood transfusion after THA

**Figure 4:**
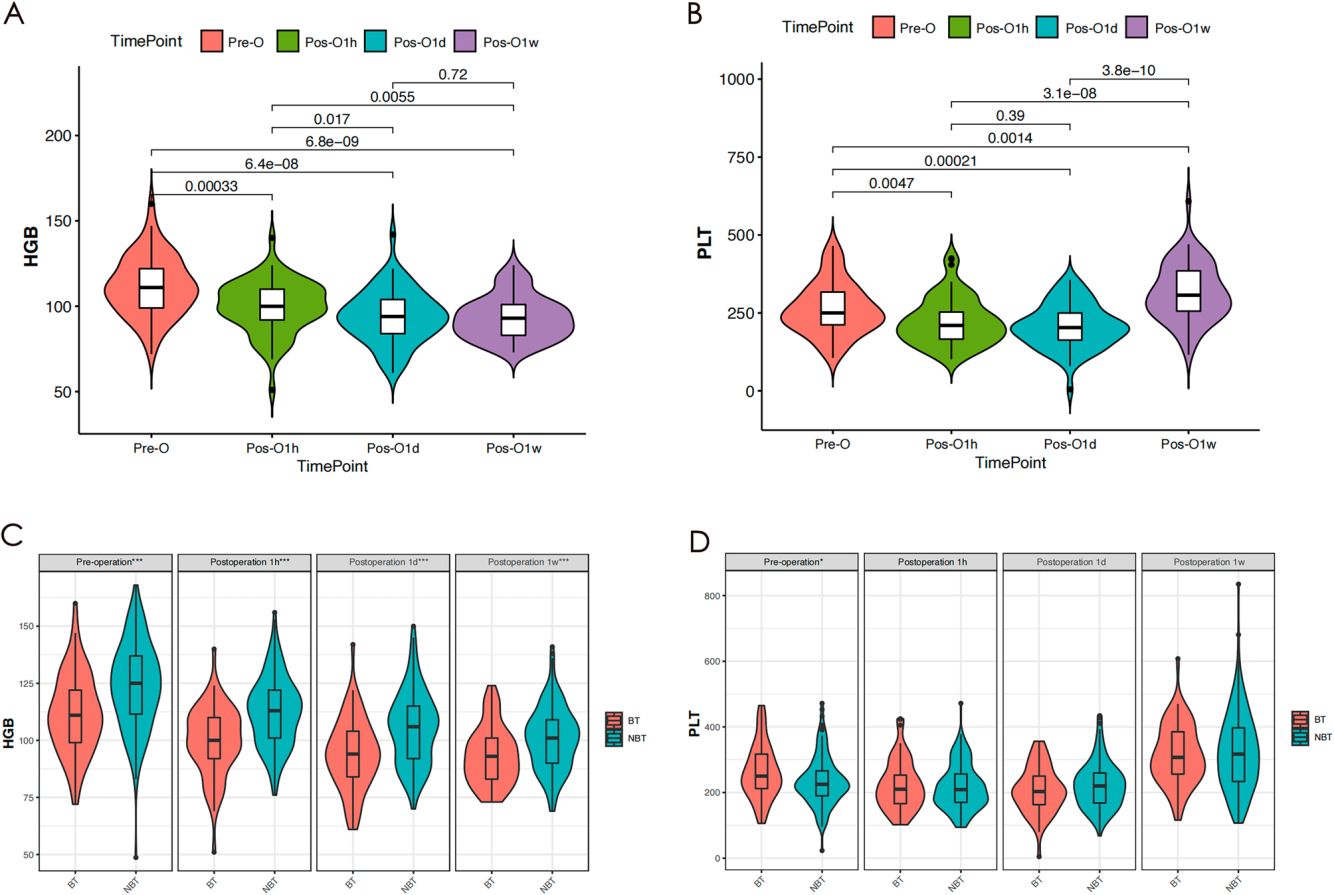
The alterations in preoperative and HGB levels at 1 h, 1 day, and 1 week among patients who received BT during the perioperative period (**A**). The alterations in preoperative and postoperative PLT levels at 1 h, 1 day, and 1 week in patients who received BT during the perioperative period (**B**). The variations in preoperative and postoperative HGB levels at 1 h, 1 day, and 1 week among patients who did and did not receive BT during the perioperative period (**C**). The variations in preoperative and postoperative PLT levels at 1 h, 1 day, and 1 week for patients who received BT during the perioperative period and those who did not (**D**). HGB: Hemoglobin, BT: Blood, PLT: Platelet.

In this study, Fig. 4A displays the alterations in preoperative and postoperative Hemoglobin (HGB) levels at 1 h, 1 day, and 1 week among patients who received Blood Transfusion (BT) during the perioperative period. Similarly, Fig. 4B portrays the modifications in preoperative and postoperative Platelet (PLT) levels at 1 h, 1 day, and 1 week in patients who received BT during the perioperative period. Furthermore, Fig. 4C illustrates the variations in preoperative and postoperative HGB levels at 1 h, 1 day, and 1 week among patients who did and did not receive BT transfusions during the perioperative period. Finally, Fig. 4D depicts the fluctuations in preoperative and postoperative PLT levels at 1 h, 1 day, and 1 week for patients who received BT transfusions during the perioperative period and those who did not.

### Results of unsupervised machine learning

**Figure 5:**
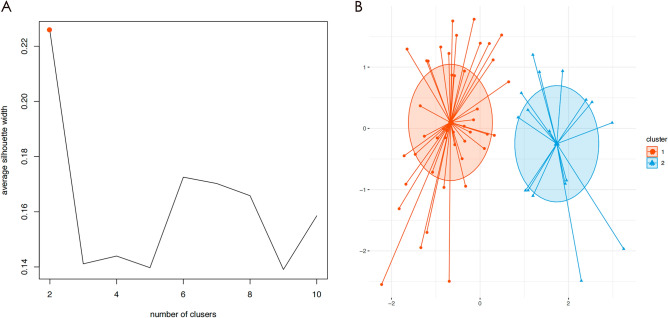
Silhouette Coefficient (SC) of K-means clustering algorithm which was determined the optimal clustering result. Peak of the broken line is the optimal value for Silhouette Coefficient (Y Axis), the optimal clustering results were equal to 2 (X Axis) (**A**). Scatter plot of 61 THA patients who received BT. Each dot in the figure represents a patient. The orange scatter represents cluster 1 and the blue scatter represents cluster 2 (**B**).

To cluster patients based on six independent predictors, we utilized the K-means algorithm. The optimal number of clusters was determined using the Silhouette Coefficient value, and Fig. 5A shows that the highest point on the broken line corresponds to the optimal value of 2 on the X-axis. This result indicates that the K-means clustering algorithm identified two clusters as optimal. The clinical data of **61** patients were successfully divided into two clusters (Fig. 5B), where each dot represents a patient, with the orange dot representing cluster 1 and the blue dot representing cluster 2. The K-means clustering algorithm's outcome is presented in Supplementary Table S1.

### Heterogeneous clinical characteristics of the patients by unsupervised machine learning

**Figure 6:**
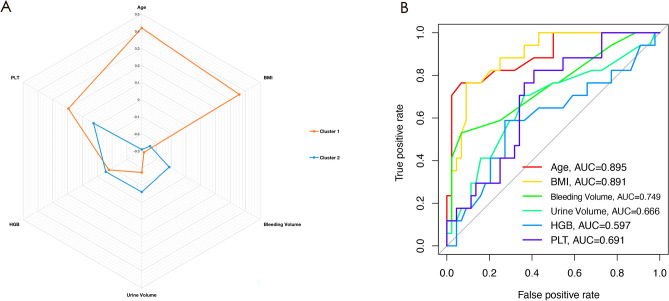
Radargram of 6 independent predictors in patients who were received blood transfusion during THA in two clusters based on K-means clustering algorithm (**A**). The AUCs of the six independent predictors for predicting Cluster 1 (**B**). BMI: Body Mass Index, HGB: Hemoglobin, PLT: Platelet.

**Figure 7:**
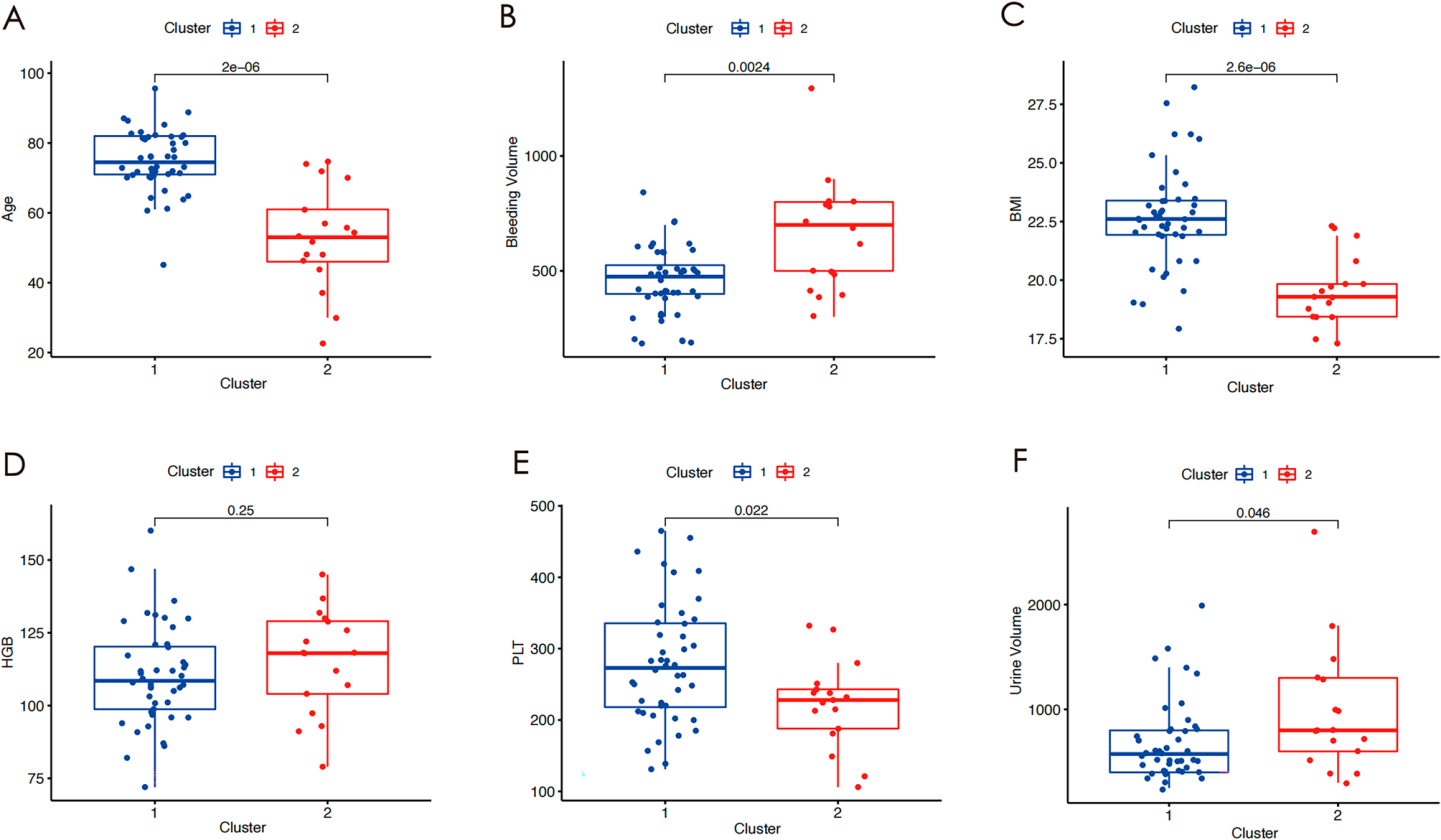
Box-line scatter plots of Age, Bleeding Volume, BMI, HGB, PLT, and Urine Volume between the two clusters (**A**–**F**). BMI: Body Mass Index, HGB: Hemoglobin, PLT: Platelet.

Table 3 presents six independent predictors of two clusters identified using the K-means clustering algorithm. As shown in Table 3, the values of Age, BMI, and PLT in cluster 1 are significantly greater than those in cluster 2 (Age: cluster 1/cluster 2 = 74.98 ± 8.81/52.94 ± 14.41, *P* < 0.001; BMI: cluster 1/cluster 2 = 23.05 ± 16.73/42.06 ± 22.84, *P* < 0.001; PLT: cluster 1/cluster 2 = 279.33 ± 83.84/221.56 ± 59.59, *p* = 0.022). In contrast, both Bleeding Volume and Urine Volume are significantly higher in cluster 2 compared to cluster 1 (Bleeding Volume: cluster 1/cluster 2 = 452.27 ± 146.53/658.82 ± 240.24, *P* = 0.002; Urine Volume: cluster 1/cluster 2 = 682.95 ± 374.31/975.29 ± 584.21, *P* = 0.046). HGB did not exhibit significant differences between the two clusters (HGB: cluster 1/cluster 2 = 110.28 ± 17.01/115.21 ± 17.40, *P* = 0.250). A radargram, depicted in Fig. 6A, was employed to visualize the heterogeneity of the two clusters. ROC curves were employed to assess the predictive performance of six independent predictive factors for Cluster 1, and the predictive efficiency was demonstrated using AUCs (based on logistic regression from the "pROC" package in R software). The AUCs of the six independent predictive factors for predicting Cluster 1 were as follows: 0.895 for age, 0.891 for BMI, 0.597 for HGB, 0.691 for PLT, 0.749 for bleeding volume, and 0.666 for Urine Volume (Fig. 6B). Figure 7A–F displays the differences in age, BMI, HGB, PLT, bleeding volume, and urine volume between the two clusters using a box-line scatter plot.

**Table 3:** Six independent predictors of two clusters identified using the K-means clustering algorithm.

Clinical Characteristics	Overall	Cluster 1	Cluster 2	*P*-Value
	(n = 61)	(n = 44)	(n = 17)	
Age				< 0.001
Mean ± SD	68.84 ± 14.54	74.98 ± 8.81	52.94 ± 14.41	
Medium[P25,P75]	72 [61, 80]	74.5 [71, 82]	53 [46, 61]	
BMI				< 0.001
Mean ± SD	21.82 ± 2.40	22.69 ± 2.11	19.56 ± 1.46	
Medium[P25,P75]	22.19 [19.83, 22.97]	22.61 [21.93, 23.39]	19.29 [18.44, 19.83]	
HGB				0.250
Mean ± SD	111.65 ± 17.26	110.28 ± 17.01	115.21 ± 17.40	
Medium[P25,P75]	111 [99, 122]	108.5 [98.75, 120.25]	118 [104, 129]	
PLT				0.022
Mean ± SD	263.23 ± 82.04	279.33 ± 83.84	221.56 ± 59.59	
Medium[P25,P75]	250 [212, 317]	273 [218, 335.5]	228 [188, 243]	
Bleeding Volume				0.002
Mean ± SD	509.84 ± 200.37	452.27 ± 146.53	658.82 ± 240.24	
Medium[P25,P75]	500 [400, 600]	475 [400, 525]	700 [500, 800]	
Urine Volume				0.046
Mean ± SD	764.43 ± 461.90	682.95 ± 374.31	975.29 ± 584.21	
Medium[P25,P75]	600 [450, 850]	575 [400, 800]	800 [600, 1300]

## Discussion

### Construction of a prediction model for blood transfusion in patients undergoing THA, based on SML and its clinical significance

LASSO regression and the Random Forest algorithm are extensively utilized for selecting predictive factors in disease data analysis. Liang et al. discovered that the platelet-to-lymphocyte ratio was an autonomous risk factor and had a correlation with the severity of AS. They used LASSO regression to conduct statistical analysis^27^. Gao et al. employed the Random Forest regression model to forecast distant metastases subsequent to stereotactic body radiation therapy for early-stage non-small cell lung cancer^28^. In this research, we employed LASSO regression and the Random Forest algorithm to identify predictive factors of patients undergoing THA who received blood transfusions in the perioperative phase. We identified 11 predictive factors that intersected, including Age, BMI, Bleeding Volume, HR, Colloid, Urine Volume, HCT, PLT, Ccr, HGB, and TXA (Fig. 2D). We constructed predictive models utilizing univariate and multivariate logistic regression techniques. Finally, six independent predictive factors were revealed, including Age, BMI, Bleeding Volume, Urine Volume, and PLT. As mentioned in the introduction section of this paper, blood transfusion is independently associated with increased morbidity and mortality in THA. The constructed model demonstrated good accuracy in predicting whether patients would receive a blood transfusion, with an AUC of 0.899 (Fig. 3F). The model allows clinicians to clinically anticipate whether patients undergoing THA will require blood transfusions during the perioperative phase using preoperative data such as Age, BMI, Bleeding Volume, Urine Volume, and PLT. With the aid of this prediction model, clinicians can effectively assess the necessity of blood transfusions in patients undergoing THA during the perioperative phase and make appropriate preparations for potential adverse effects, thereby facilitating the implementation of precision medicine.

### The orientation and interpretability of unsupervised machine learning

This study employed unsupervised machine learning, specifically K-means clustering, to categorize patients undergoing total hip replacement who received blood transfusions in the perioperative phase. Our decision to utilize K-means clustering was rooted in its suitability for this particular context. K-means clustering has several advantages that make it well-suited for our research objectives. Firstly, it is computationally efficient and capable of handling large datasets, a vital consideration in clinical studies where data can be extensive. Secondly, it is relatively straightforward to implement, making it accessible to researchers without extensive machine learning expertise^29^.

In our analysis, K-means demonstrated remarkable data separation between the two patient clusters, as evident in the radargram (Fig. 6A). This separation allows for the identification of distinctive patient groups with varying clinical characteristics, which can have significant implications for personalized treatment plans. This methodological choice aligns with the ultimate goal of our research, which is to seamlessly integrate inherently diverse and heterogeneous clinical data without supervision^30^.

While we have discussed the strengths of K-means clustering, it is important to acknowledge its limitations. K-means is sensitive to the initial cluster centers, and different starting points may lead to different cluster results^31^. This challenge underscores the importance of careful preprocessing and initialization strategies, which we meticulously addressed in our study. Furthermore, K-means clustering may not be the optimal choice for datasets with irregularly shaped or non-convex clusters, and in such cases, alternative approaches like density-based clustering or hierarchical clustering may be considered.

This emphasis on unsupervised machine learning (UML) in our study is a deliberate choice. UML prioritizes the intrinsic characteristics of the data, enabling us to delve deeper into the fundamental features and emphasize the diversity of clinical traits that are relevant to medical research hypotheses^32^. By doing so, we uncover latent structures within our data that might not be apparent using traditional, supervised machine learning methods. This approach not only enriches our understanding of patient diversity but also contributes to the evolving landscape of medical research.

In conclusion, the choice of K-means clustering in our study was driven by its appropriateness for our research objectives and data characteristics. It is important to consider the specific research goals and data properties when selecting the most suitable method. The application of UML techniques, as seen in our research, has the potential to yield valuable insights in the field of medical research and can serve as a powerful tool for uncovering hidden patterns within clinical data. Future research may further explore the use of alternative clustering methods and deep learning techniques to enhance our understanding of patient diversity and improve medical decision-making.

### Heterogeneity of clinical characteristics based on the classification of unsupervised machine learning

Using the clustering outcomes, we compared the variation of six independent predictors between the two clusters to confirm the effectiveness of unsupervised machine learning (UML) clustering. As demonstrated in Fig. 6A, the dissimilarity of Age, BMI, PLT, Bleeding Volume, and Urine Volume between the two clusters is substantial and can effectively differentiate between them (Fig. 6B). UML can efficiently categorize patients based on their clinical characteristic heterogeneity, offering an understandable and significant classification of a diverse cohort^33^. In our research, this intelligibility was reflected in the heterogeneity of Age, BMI, PLT, Bleeding Volume, and Urine Volume.

To conclude, UML was able to sufficiently classify 61 patients undergoing total hip replacement who received blood transfusions in the perioperative phase into two clusters according to their fundamental clinical attributes. A thorough comprehension of patient heterogeneity can detect and manage complications during the perioperative period and provide beneficial guidance for the implementation of precision medicine.

### Clinical significance of the combination of supervised machine learning and unsupervised machine learning

In this study, we developed an SML predictive model to assess the need for blood transfusion in THA patients, identifying six predictive factors: Age, BMI, Bleeding Volume, Urine Volume, PLT, and HGB. Based on the SML model, attention should be focused on these six BT-related predictive factors during the perioperative management of THA patients, aiming to enhance preoperative preparation and improve the quality of perioperative care. Furthermore, to explore clinical heterogeneity in THA patients requiring BT, we applied the UML algorithm based on K-means clustering. The results indicate that the UML algorithm clustered THA patients into two groups based on these six predictive factors. Patients in Cluster 2 exhibited significantly higher Bleeding Volume and Urine Volume compared to Cluster 1, while their PLT was significantly lower (Table 3). Bleeding Volume, Urine Volume, and PLT precisely reflect crucial indicators of effective circulating blood volume in THA patients. This suggests that patients in Cluster 2 may have a poorer effective circulating blood volume. The above results enlighten us that, in the perioperative management of THA patients requiring blood transfusion, there is a subset with inadequate circulating blood volume characterized by high Bleeding Volume and Urine Volume and low PLT. Such patients in this cluster may have unstable hemodynamics, warranting special attention from clinicians. In the perioperative management of patients, clinicians should first evaluate the likelihood of blood transfusion based on the six predictive factors from the SML model, improving preoperative preparation and enhancing perioperative care quality. Subsequently, post-blood transfusion in THA patients, attention should be directed to Bleeding Volume, Urine Volume, and PLT using the UML algorithm results, allowing timely medical intervention to prevent adverse perioperative events.

### Surgeon's influence and limitations

In this section, we will discuss the study’s limitations, with a specific focus on how a surgeon's individual preferences and prior experiences may have impacted our results. Factors specific to the surgeon can introduce data variability and influence result interpretation^34^. Our research constructed a predictive model for blood transfusion and primarily examined the clinical diversity among patients undergoing THA surgery and receiving blood transfusions. Nevertheless, it is essential to recognize that the surgeons' distinct surgical techniques, implant preferences, and perioperative practices, influenced by their experiences, can unintentionally contribute to clinical diversity^35^. The influence of surgeon-specific factors on our findings constitutes a noteworthy limitation. Future research should delve more comprehensively into this aspect, possibly through collaborations with surgical teams and additional data gathering. The development of standardized procedures and guidelines for specific surgical aspects could potentially mitigate the impact of surgeon-specific variables^3^. In conclusion, while our study illustrates patient clinical diversity, we recognize the constraints linked to surgeon preferences and experiences. Addressing these limitations and integrating these factors into data analysis and interpretation is crucial for a more profound comprehension of patient outcomes.

Additionally, there are other limitations in our current study that need to be acknowledged. First, the participants were recruited exclusively from a single center. Second, given that this is a retrospective study, there is the potential for selection bias. Furthermore, it is possible that the surgeon's individual preferences and prior experiences could have influenced the study's outcomes.

## Conclusion

Age, BMI, PLT, HGB, Bleeding Volume, and Urine Volume were identified as independent predictors of whether a THA patient requires a blood transfusion. The predictive model constructed based on these six independent predictors displayed remarkable predictive performance. Unsupervised machine learning (UML) can offer a clear and meaningful classification of a diverse cohort of THA patients who received BT.

### Supplementary Information

Supplementary Information 1.

Supplementary Information 2.

Supplementary Information 3.

## Data Availability

The original contributions presented in this study are available in the article/supplementary material. More inquiries can be directed to the corresponding authors.

## Abbreviations

BT: Blood transfusions
THA: Total hip arthroplasty
ML: Machine learning
SML: Supervised machine learning
UML: Unsupervised machine learning
RF: Random forest
LASSO: Least absolute shrinkage and selection operator
BMI: Body mass index
HGB: Hemoglobin
PLT: Platelet
ASA: American society of anesthesiologists
CVD: Cardiovascular disease
COPD: Chronic obstructive pulmonary disease
PAH: Pulmonary arterial hypertension
TXA: Tranexamic acid
SP: Systolic pressure
DP: Diastolic pressure
HR: Heart rate
RBC: Red blood cell
HGB: Hemoglobin
HCT: Hematocrit
PLT: Platelet
TBIL: Total bilirubin
TP: Total protein
ALB: Albumin
ALT: Alanine aminotransferase
AST: Aspartate aminotransferase
CREA: Creatinine
BUN: Blood urea nitrogen
Cys-C: Cystatin C
Ccr: Creatinine clearance rate
Hs-CRP: Hypersensitive C-reactive protein
PT: Prothrombin time
APTT: Activated partial thromboplastin time
FIB: Fibrinogen
DD: D-Dimer
SC: Silhouette coefficient
AUC: Area under the curve
